# Preduodenal Portal Vein with Situs Inversus Totalis causing Duodenal Obstruction

**DOI:** 10.21699/ajcr.v7i3.435

**Published:** 2016-06-15

**Authors:** Flavia D’souza, Amol Nage, Pradnya Bendre

**Affiliations:** Department of Pediatric Surgery, Bai Jerbai Wadia Hospital, Mumbai

**Keywords:** Duodenal atresia, Preduodenal portal vein, Situs inversus, Newborn

## Abstract

Congenital duodenal obstruction sometimes may be secondary to unusual entities like preduodenal portal vein (PDPV) the identification of which is very important to avoid inadvertent injury or incorrect surgery. A 6-day old neonate presented with congenital duodenal obstruction. Investigations revealed situs inversus totalis with many congenital cardiovascular anomalies. At operation preduodenal portal vein and malrotation were found. Correction of malrotation and bypass duodeno-duodenostomy were done.

## CASE REPORT

A 6-day-old preterm girl, weighing 1400 g, presented with bilious vomiting, dehydration, lethargy, and poor sucking. On examination, she was dehydrated with scaphoid abdomen. Other systemic examination was normal. Babygram showed dextrocardia, with liver on the left and reversal of stomach bubble. Ultrasonography (USG) of abdomen showed situs inversus totalis with duodenal atresia. Echocardiography findings were of dextrocardia, small atrial septal defect (ASD) with left to right shunt, atrial situs solitus, bilateral superior vena cava (SVC) and interrupted inferior vena cava (IVC) with azygous continuation into left SVC draining into dilated coronary sinus.

On exploration, there was dilated first part of the duodenum with portal vein compressing duodenum without intrinsic obstruction (Fig. 1). Associated malrotation of gut was also present along with situs inversus. Correction of malrotation with side-to-side duodeno-duodenostomy, bypassing preduodenal portal vein, was done. There was no associated duodenal web or atresia. Patient had an uneventful recovery and doing fine at follow-up.

**Figure:**
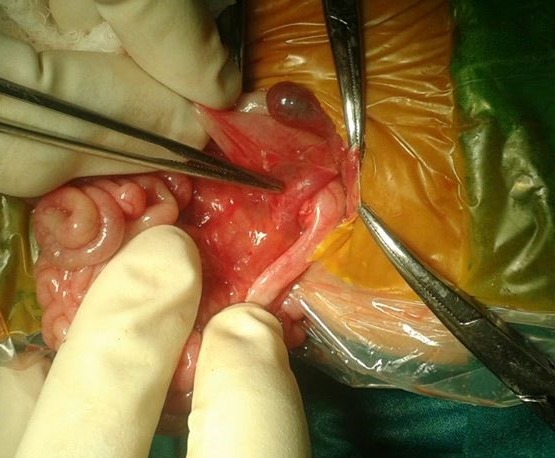
Figure 1:Preduodenal portal vein.

## DISCUSSION

Splenic malformation, cardiac defects, malrotation of gut and duodenal obstruction are known associations with situs inversus. Besides duodenal atresia, preduodenal portal vein (PDPV) may be the association or cause of duodenal obstruction.[1-4] The other common associations are cardiac anomalies and annular pancreas. [5-7] Congenital cardiac anomalies are present in about 5-10% of patients with situs inversus.[6]

PDPV is a rare anomaly in which the portal vein passes anterior to the duodenum rather than posteriorly, mostly seen in situs inversus. Isolated PDPV which may remain asymptomatic till adulthood is an incidental finding but rarely can cause duodenal obstruction.[2, 3] It is extremely important to be aware of variations in portal vein and mesenteric arteries and veins in situs inversus if surgical procedures are planned. The latter vessels are also useful as a marker for malrotation. Abdominal doppler ultrasound study may help diagnose these vascular anomalies.

Postnatal presentation may be indistinguishable from duodenal atresia but association of situs inversus should raise the suspicion. Intraoperatively if PDPV is seen it is mandatory to rule out intrinsic obstruction which is more common than PDPV itself causing obstruction. If PDPV is not causing obstruction, intraluminal web or duodenal atresia may be the cause of obstruction. However, if PDPV is the cause of obstruction, then duodeno-duodenostomy anterior to the portal vein is the procedure of choice.[5-7]

## Footnotes

**Source of Support:** Nil

**Conflict of Interest:** None declared

